# Alterations of Serum Bile Acid Profile in Patients with Crohn's Disease

**DOI:** 10.1155/2022/1680008

**Published:** 2022-10-03

**Authors:** Rui Sun, Jingjing Jiang, Ling Yang, Lu Chen, Hong Chen

**Affiliations:** ^1^School of Medicine, Southeast University, Nanjing, China; ^2^Department of Gastroenterology, Zhongda Hospital of Southeast University, Nanjing, China

## Abstract

**Background:**

Bile acid (BA) metabolism may be influenced by gut dysbiosis and alterations of intestinal epithelium in patients with Crohn's disease (CD). Here, we aimed at investigating the alterations of serum BA profile in CD patients and analyzing the correlation between BAs and CD disease activity.

**Methods:**

A total of 62 CD patients (29 active and 33 remission) and 33 healthy volunteers (HVs) were enrolled in this retrospective study. Serum BA profiles were measured by liquid chromatography-tandem mass spectrometry.

**Results:**

Levels of primary BAs components, including cholic acid (CA) and chenodeoxycholic acid (CDCA), showed no significant difference when compared with HVs. Secondary BAs (SBA) were significantly decreased in CD patients compared with HVs. Importantly, the deoxycholic acid (DCA) and glycodeoxycholic acid (GDCA) levels were significantly lower in CD active than in CD remission patients. The DCA/(DCA + CA) ratio was also decreased in CD active patients than in CD remission patients while the LCA/(LCA + CDCA) ratio showed no difference between them. Principal component analysis also indicated a clear separation among the three groups, with a total variance of 93.43%. The correlation analysis showed that the SBA, DCA, GDCA levels, and DCA/(DCA + CA) ratio had an inverse relationship with Crohn's Disease Activity Index.

**Conclusion:**

The BA profile exhibits significant alterations in CD patients. The SBA, DCA, GDCA levels, and DCA/(DCA + CA) ratio were significantly decreased in CD active patients. The DCA/(DCA + CA) ratio had an inverse correlation with CD disease activity.

## 1. Introduction

Crohn's disease (CD) represents a relapsing inflammatory bowel disease (IBD) featuring chronic abdominal pain, diarrhea, obstruction, and perianal disease [1]. While previous studies demonstrated the genetic, environmental, microbial, and immunological factors as triggers of CD [2, 3], its precise etiology remains unknown. Since gut dysbiosis is closely associated with CD pathogenesis [4], the gut microbiota-bile acid (BA) axis can be a promising research area in CD patients.

In the liver, cholesterol 7*α*-hydroxylase (CYP7A1) catalyzes the transformation of cholesterol to primary BAs, including cholic acid (CA) and chenodeoxycholic acid (CDCA) [5]. Primary BAs are conjugated with glycine or taurine before being stored in the gallbladder. Particularly after dinner when the gallbladder contracts, BAs are secreted into the duodenum. It is estimated that 95% of the primary BAs are reabsorbed in the ileum, within the frame of enterohepatic circulation [6, 7]. The remaining primary BAs are processed into secondary BAs (SBA) by deconjugation and 7*α*-dehydroxylate of the gut microbiota, involving deoxycholic acid (DCA), ursodeoxycholic acid (UDCA), and lithocholic acid (LCA) [8].

Several previous studies analyzed the BA concentration and gut microbiota composition to conduct targeted metabolomics investigations in IBD patients. For example, Duboc et al. revealed a significantly increased ratio of conjugated BAs, paralleled by decreased levels of SBAs in fecal samples of active IBD patients [9]. Moreover, Murakami et al. demonstrated that reduced *Clostridium* subcluster XIVa, serving for 7*α*-dehydroxylation, is positively correlated with a low DCA/(DCA + CA) ratio [10]. In addition, Sinha et al. documented a positive correlation of *Firmicutes ruminococcaceae* with the SBA levels (such as DCA and LCA) in ulcerative colitis patients [11]. Altogether, these findings indicate that BA dysmetabolism could be associated with gut dysbiosis in IBD patients.

Other studies have shown that due to decreased diversity of microbiota, SBAs could be reduced in CD patients [9, 11–13]. It has been proved that exogenous supplementation of BAs could relieve the intestinal inflammation and regulate T cell homeostasis in the murine colitis model [11, 14]. It is reasonable to take BAs into the management of CD. However, to our best knowledge, no study investigated the alterations of BAs and the correlation between BAs and CD activity in Chinese patients. Our retrospective study aimed to measure the BA levels and evaluate the clinical significance of inflammatory activity in CD.

## 2. Methods

### 2.1. Subjects

A total of 62 CD patients were consecutively enrolled in this study. All the subjects were hospitalized in the Department of Gastroenterology, Zhongda Hospital of Southeast University, from August 2020 to August 2021. The CD diagnostic criteria were corroborated with clinical, imaging, endoscopic, and histological manifestations based on the European Crohn's and Colitis Organisation consensus [15]. The exclusion from the study was decided when one of the criteria: (a) liver or biliary tract complications, (b) elevated liver enzymes, and (c) antibiotics taken in the past 3 months was met. The location of the disease was defined as ileal (L1), colonic (L2), and ileocolonic (L3) as previously reported by the Montreal classification [16]. The disease activity of CD was evaluated by the Crohn's Disease Activity Index (CDAI). Additionally, the control group (*n* = 33) comprised clinically healthy subjects from the Medical Examination Center of the Zhongda Hospital and had no prior history of liver dysfunction or antibiotics taken over the past 3 months. The study was approved by the Ethics Committee of Zhongda Hospital (2021ZDSYLL297-P01).

### 2.2. Research Methods

Serum samples were collected from all subjects after overnight fasting. BA levels were measured by liquid chromatography-tandem mass spectrometry. Other laboratory data, such as C-reactive protein (CRP) and erythrocyte sedimentation rate (ESR) were obtained and documented at the clinical laboratory of the Zhongda Hospital. Data collected from the CD cohort includes age, gender, disease duration, site of disease, and medications. Furthermore, the CDAI score was calculated to differentiate CD patients from active to remission period and the CDAI score <150 was considered as the remission period [17].

### 2.3. Statistical Analysis

Statistical analysis was performed with SPSS Statistics 23.0 and GraphPad Prism 8.4.0 software. The analysis involved tests of normality and homogeneity of variance. The differences between groups were assayed for parametric by one sample *t*-test, expressed as mean ± standard deviation (SD). Otherwise, the Mann–Whitney *U* test was employed for comparisons of nonparametric data, expressed as median (interquartile range). The correlation was assessed by the Pearson's correlation coefficient or nonparametric Spearman's rank correlation coefficient. Metabolomic analysis was performed by heatmaps and principal component analysis (PCA) to assess the separation between groups using R software (version 3.6). Variables were mean-centered and divided by the SD before statistical analysis in the heatmap. In all statistical tests, *P* < 0.05 was considered as statistically significant.

## 3. Results

### 3.1. Characteristics of Study Subjects

In this retrospective study, we included 62 CD patients and 33 controls out of which 29 were CD active patients (18 males and 11 females, 43.7 ± 16.3 years) and 33 CD remission patients (18 males and 15 females, 40.0 ± 14.3 years). Healthy volunteers (HVs) involved 20 males and 13 females, with an average age of 46.0 ± 10.9 years (Table 1). No significant difference between CD active and remission patients with respect to the duration (in years), surgical history and site of disease (*P* > 0.05) was documented. About 3% of CD active patients, but no CD remission patients were administered with corticosteroid therapy. 72% of CD active and 85% of CD remission patients were treated with biological agents, such as anti-tumor necrosis factor *α* (anti-TNF *α*) or anti-*α*4*β*7 integrin antibodies, but there was no significant difference in the exposure to biologics between CD active and remission patients. CD active patients had significantly higher CRP and ESR levels than CD remission patients (*P* < 0.001), and CDAI scores could differentiate them from active to remission period.

### 3.2. Analysis of Serum Bile Acid Levels

**Figure 1:**
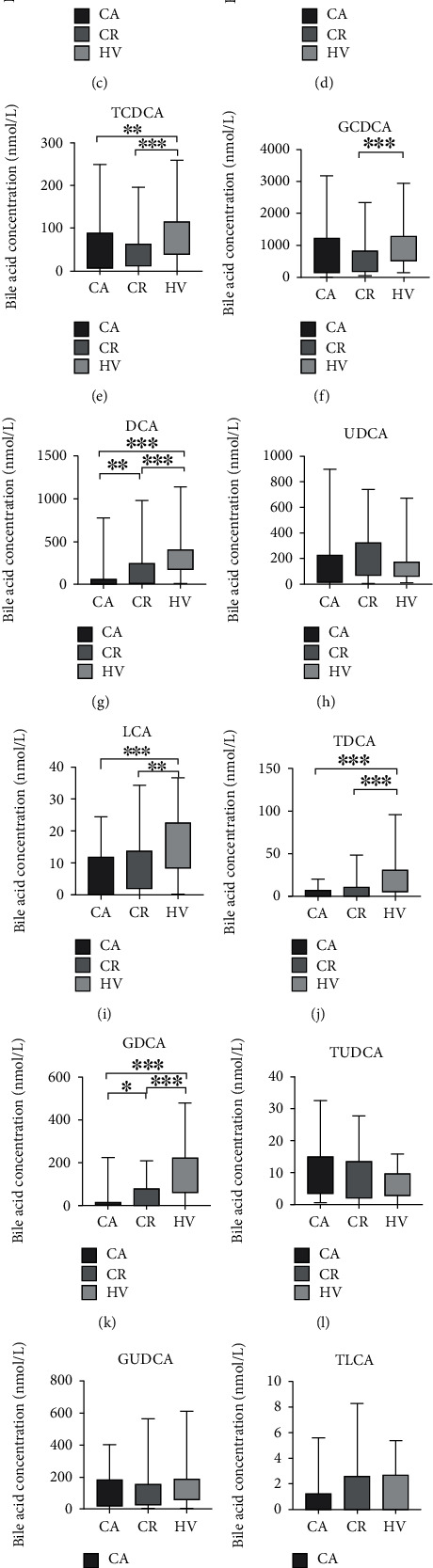
Serum bile acid profile in CD patients and healthy controls. The bile acid concentration was determined in CD active (CA), CD remission (CR), and HV groups. Differences were analyzed with Mann–Whitney *U* test. ∗*P* < 0.05, ∗∗*P* < 0.01, and ∗∗∗*P* < 0.001. CA: cholic acid; CDCA: chenodeoxycholic acid; TCA: taurocholic acid; GCA: glycocholic acid; TCDCA: taurochenodeoxycholic acid; GCDCA: glycochenodeoxycholic acid; DCA: deoxycholic acid; UDCA: ursodeoxycholic acid; LCA: lithocholic acid; TDCA: taurodeoxycholic acid; GDCA: glycodeoxycholic acid; TUDCA: tauroursodeoxycholic acid; GUDCA: glycoursodeoxycholic acid; TLCA: taurolithocholic acid; GLCA; glycolithocholic acid.

**Figure 2:**
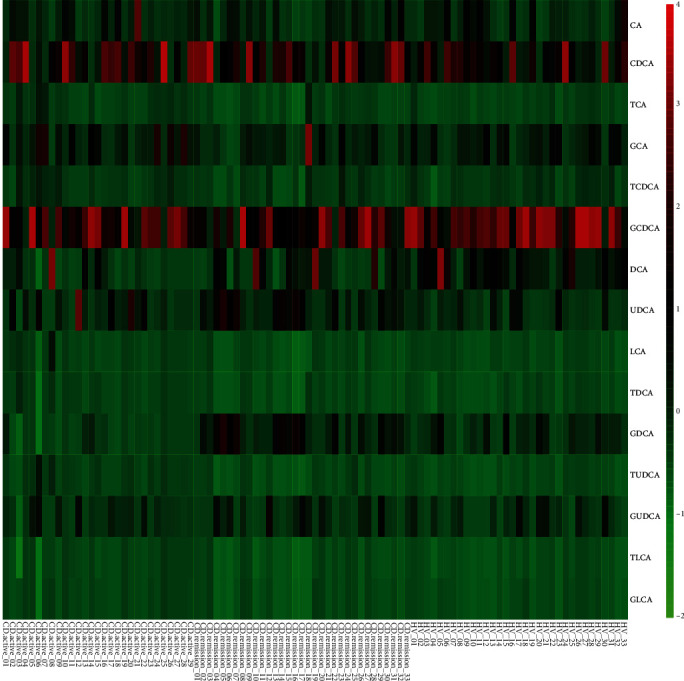
A heatmap analysis of serum metabolites among all participants. Groups were shown at the bottom of the figure as CD active (*n* = 29), CD remission (*n* = 33), and HVs (*n* = 33). Green indicates a lower level than the average and red indicates a higher level than the average. Gradient changes of bile acids are reflected through colors in the heatmap.

Concentrations of total and individual BAs were detected in different groups (Table 2; Figure 1). Generally, concentrations of total BAs were analyzed in the entire population and serum SBAs showed great differences among CD active, CD remission, and HV groups (Table 2). Each BA concentration was taken into analysis in detail (Figure 1). No significant difference in primary BAs (CA and CDCA) among the CD active, CD remission, and HV groups was observed (Figures 1(a) and 1(b)). In the primary conjugated BAs, bound to glycine or taurine, the TCA, GCA, TCDCA, and GCDCA levels were reduced in CD remission patients when compared with the HVs (Figures 1(c)–1(f)). In addition, the TCA and TCDCA levels were decreased in CD active patients (Figures 1(c) and 1(e)). With respect to SBAs, both DCA and LCA levels were lower in CD patients than in the controls [Figures 1(g) and 1(i)]. Importantly, the DCA level in CD active patients was decreased when compared with CD remission patients (*P* < 0.01; Figure 1(g)). There was no significant difference in UDCA and its conjugated glycine or taurine among the three groups [Figures 1(h), 1(l), and 1(m)]. Meanwhile, TDCA, GDCA, and GLCA levels in CD patients also decreased when compared to the controls (Figures 1(j), 1(k), and 1(o)). And the GDCA level was significantly lower in CD active patients (*P* < 0.05; Figure 1(k)). In addition, according to the heatmap, DCA and GDCA levels were reduced in CD active patients when compared with CD remission patients and healthy controls (Figure 2), consistent with data presented in Figure 1.

**Figure 3:**
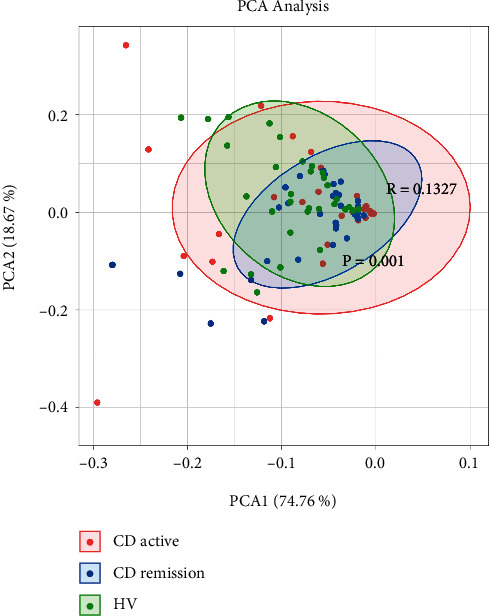
Principal component analysis (PCA) of bile acid profile among three groups. CD active patients (red), CD remission patients (blue), and HVs (green) are shown.

In order to further explore the metabolic differences within these samples, PCA analysis was conducted. Our analysis indicates a clear separation among the CD active, CD remission, and HVs when involving all identified BAs (Figure 3). The variability of PC1 and PC2 was 74.76% and 18.67%, respectively, obtained as 93.43% of total variance.

**Figure 4:**
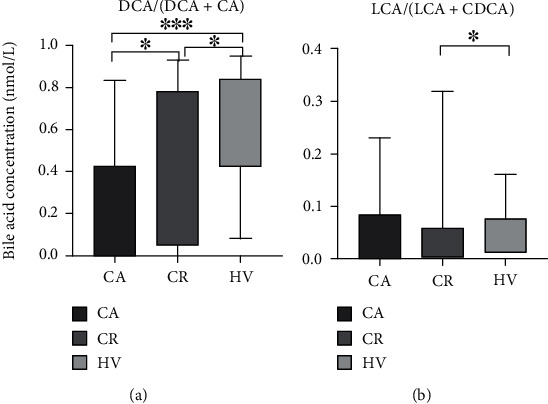
Comparison of serum secondary/(primary + secondary) BA ratios in the entire population. ∗*P* < 0.05, ∗∗*P* < 0.01, and ∗∗∗*P* < 0.001.

Having delineated that gut microbiota contributes to the transformation from primary to SBAs via 7*α*-dehydroxylation [10], we sought to calculate the secondary/(primary + secondary) BA ratios before investigating the correlation with the severity of CD activity. We found that DCA/(DCA + CA) ratio featured a significant difference among three groups, while the LCA/(LCA + CDCA) ratio only decreased in CD remission patients but not in the CD active patients (Figure 4).

### 3.3. Correlation between BA Levels and CD Disease Activity

**Figure 5:**
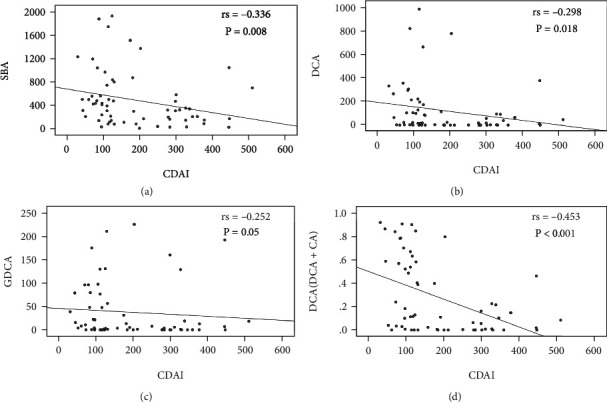
Correlation of laboratory biomarkers and bile acid markers with disease activity in CD patients.

As several BAs were significantly altered between CD active and remission patients, we analyzed the correlation of them with CDAI scores (Figure 5). SBAs were negatively correlated with CDAI scores from the Spearman's correlation analysis (*r*_s_ = −0.336, *P* = 0.008), shown in Figure 5(a). Meanwhile, serum DCA and GDCA levels in CD patients had a negative correlation with CDAI scores Figures 5(b) and 5(c)]. An inverse relationship between the DCA/(DCA + CA) ratio and CDAI scores (*r*_s_ = −0.453, *P* < 0.001) was also documented [Figure 5(d)].

## 4. Discussion

Our investigations revealed that significant alterations of the serum BA profile in CD patients. SBAs, such as DCA and GDCA, were significantly lower in CD active than in CD remission patients. Furthermore, the DCA/(DCA + CA) ratio was also decreased in CD active patients. The heatmap substantiated these findings and the PCA analysis revealed a clear separation within these groups. In addition, the DCA/(DCA + CA) ratio had an inverse correlation with the clinical disease activity of CD patients.

Previous studies had reported that the alterations of both serum and fecal BA concentrations in IBD patients. Duboc et al. found that low SBAs could be associated with the impaired gut microbiota [9]. However, they did not analyze each BA proportion in detail. In the current study, from the 15 types of BAs taken under investigation, the SBAs displayed low levels in the CD patients. Furthermore, Roda et al. reported the physiological concentration of SBAs was restored in CD patients subjected to anti-TNF*α* therapy. Yang et al. investigated the association of decreased fecal BAs and gut microbiota dysregulation and the mechanism of inflammatory responses through BA-activated receptors in patients with ulcerative colitis [18]. However, they did not evaluate the association between BA concentration and disease activity.

Our study revealed a significant decrease in SBAs in CD patients. SBAs are considered to play an anti-inflammatory role in IBD patients [11, 19]. The high levels of proinflammatory cytokines in the IBD were associated with the disruption of the epithelial barrier [20, 21]. In this scenario, SBAs can prevent the apoptosis orchestrated by cytokines in the epithelium and therefore relieve the mucosal inflammation [22, 23]. Furthermore, SBAs can also regulate the host immune response by maintaining the balance between helper T cells and regulatory T cells [24]. In this study, we found that SBAs, including DCA and LCA, were lower in CD patients than in HVs. The DCA level was significantly lower in CD active than in CD remission patients, while LCA showed no difference between them. In addition, DCA/(DCA + CA) ratio is associated with the 7*α*-dehydroxylate of *Clostridium* subcluster XIV*α* and represents the conversion from primary to SBAs [10]. Here, we also demonstrated that DCA/(DCA + CA) ratio was significantly lower in CD active patients compared with CD remission patients.

However, the association between the BA profile and disease activity still remains elusive. Given the alterations of several BAs among the CD active, CD remission, and healthy population, we evaluated the correlation of these indicators and disease activity. SBAs had a negative relationship with CDAI scores (*r*_s_ = −0.336). Furthermore, the DCA and GDCA levels showed an inverse correlation with CD activity (*r*_s_ = −0.298 and −0.258, respectively). The DCA/(DCA + CA) ratio was also negatively correlated with CDAI scores (*r*_s_ = −0.453). Overall, it seems likely that increased inflammatory responses tend to decrease SBA levels in CD patients with higher CDAI scores.

It is fair to say that our study also has some limitations. First, while the sample size was small, we could still observe differences of the BA profile in CD patients compared with healthy subjects. But a larger number of samples will further strengthen these findings. Second, despite the fact that alterations of serum and fecal BAs were consistent with the previous literature, we think that involvement of the fecal BA profile would yield stronger data. Lastly, as a retrospective study, we did not involve the microbiome data to explore the relationship between gut dysbiosis and BA alterations. Our colleagues will further take a prospective study to investigate the relationship of them to better understand the role of BAs for a promising treatment strategy.

To summarize, this study indicates the alterations of the serum BA profile. The DCA/(DCA + CA) ratio may be a potential biochemical signature for delineation of the intestinal inflammation in CD patients.

## Figures and Tables

**Table 1:** Characteristics of patients with Crohn's disease and healthy volunteers.

Characteristics		CD active (*n* = 29)	CD remission (*n* = 33)	HVs (*n* = 33)
Age (years)		43.7 ± 16.3	40.0 ± 14.3	46.0 ± 10.9
Gender (male/female)		18/11	18/15	20/13
Disease duration (years)		5 (1–8)	3 (1–6)	—
Surgical history, *n* (%)		12 (41)	18 (55)	—
Site of disease, *n* (%)	Ileal (L1)	7 (24)	13 (39)	—
	Colonic (L2)	3 (10)	2 (6)	—
	Ileocolonic (L3)	19 (66)	18 (55)	—
Medications, *n* (%)	Mesalazine	13 (45)	6 (18)	—
	Corticosteroids	1 (3)	0 (0)	—
	Azathioprine	5 (17)	5 (15)	—
	Biologics	21 (72)	28 (85)	—
CRP (mg/dL), median (IQR)		29.6 (13.3–56.7)∗	0.9 (0.8–1.3)	
ESR (mm/hour), median (IQR)		36.0 (18.5–66.5)∗	6.0 (3.0–12.0)	
CDAI, median (IQR)		298.8 (207.1–352.1)∗	97.2 (76.9–117.3)	

CD: Crohn's disease; HV: healthy volunteer; CRP: C-reactive protein; ESR: erythrocyte sedimentation rate; CDAI: Crohn's disease activity index; IQR: interquartile range. ∗*P* < 0.05 between groups on Mann–Whitney *U* test.

**Table 2:** Concentrations of different bile acids in the entire population.

BAs (nmol/L)	CD active (*n* = 29)	CD remission (*n* = 33)	HVs (*n* = 33)
Total BAs	2076.5 (563.3–4168.7)	1530.9 (1084.2–3135.0)∗	2978.2 (1896.1–4266.2)
Primary BAs	1451.2 (331.1–3776.4)	1044.5 (764.6–2451.5)∗	2088.8 (1249.2–2908.0)
Secondary BAs	217.0 (152.9–473.5)^**#**^∗∗	480.1 (233.8–820.4)∗	795.3 (460.3–1175.9)
Conjugated BAs	675.4 (262.5–2118.7)	936.2 (394.0–1220.7)∗∗	1479.0 (871.4–2446.4)
Unconjugated BAs	491.9 (138.3–1969.5)	867.4 (360.9–1826.4)	1295.9 (571.1–1618.4)

Results are shown as median (interquartile range). Differences were analyzed with Mann–Whitney *U* test. ^**#**^Comparison of CD remission patients to CD active patients. ∗Comparison of HVs to CD active or remission patients, ∗∗*P* < 0.01.

## Data Availability

Data supporting this research article are available from the corresponding author or first author on reasonable request.
